# Some Every-day Points in Veterinary Jurisprudence

**Published:** 1895-08

**Authors:** Joseph N. B. Rawle


### SOME EVERY-DAY POINTS IN VETERINARY JURISPRUDENCE.

BY JOSEPH N. B. RAWLE, L.B., M.D.S., V.S.

WARRANTIES made upon the sale of chattels are with some exceptions express and not implied contracts, and must be made at the sale or prospectively in view of it. Vendors warrant the title to the goods sold if in possession, otherwise not. Vendors who do not claim to sell goods as their own, but of another, do not warrant longer than the time the purchase money remains in the hands of the vendor.

It is unnecessary to use the word "warrant," for no particular set of words constitutes a valid warranty, any affirmation respecting the thing to be sold which the purchaser may rely upon as a fact and not the expression of an opinion is sufficient, as negotiating the sale of a horse, "I promise you he is sound," or "you may depend upon it the horse is perfectly quiet and free from vice," or that the horse "was not lame, and that he would not be afraid to warrant it sound every way as far as he knew," or "well, the cow is all right," or that the jack "is a good and sure foal-getter." If a horse is purchased to use in harness and the vendor says he is "all right," this a warranty of his soundness and of his fitness for use in harness."

Any mere commendation by the vendor is not a warranty, unless it is shown that both parties understood it to be one.

A general warranty relates to the general character of the thing warranted, as a horse is free from disease or vice. In such a warranty, although it may be in writing, but if there is any defect which is pointed out to the buyer, or plainly visible, the warranty will not extend to it. Therefore, if an animal is lame or diseased in the eyes, nose, or feet, and the purchaser is informed of that fact and a warranty given that it is sound, will in effect only be that it is sound except those defects; the vendee or buyer to guard against that must obtain a special warranty, as the lameness of the animal will not be permanent, but the vendor must not use any artifice or say or do anything to divert the eye or obscure the observation of the buyer, or to otherwise mislead him even in relation to open defects. If he does so, he is guilty of fraud. If the vendee is unable to discover the defects for want of skill or of eyesight, a general warranty is sufficient.

These few remarks on warranty are for the busy veterinary surgeon who may be called upon for his opinion in relation to the law as now applied in the sale of horses; it is only superficial and not extended, but each of the questions therein passed upon have come within judicial decisions of this country and Europe, and have frequently been averted to in the trial of cases which have come under the notice of the writer. At some future time I shall compile for this JOURNAL a more extended article, which I hope will be of some little service to veterinarians.

